# Traditional and complementary medicine (TCM) usage and its association with Patient Assessment of Chronic Illness Care (PACIC) among individuals with metabolic syndrome in primary care

**DOI:** 10.1186/s12906-021-03493-x

**Published:** 2022-01-13

**Authors:** Nor Farha Basri, Anis Safura Ramli, Mariam Mohamad, Khairatul Nainey Kamaruddin

**Affiliations:** 1 Department of Primary Care Medicine, Faculty of Medicine, Universiti Teknologi MARA (UiTM), Selayang Campus, Jalan Prima Selayang 7, 68100 Batu Caves, Selangor Malaysia; 2 Institute of Pathology, Laboratory and Forensic Medicine (I-PPerForM), Universiti Teknologi MARA (UiTM), Sungai Buloh Campus, Jalan Hospital, 47000 Sungai Buloh, Selangor Malaysia; 3 Department of Population Health and Preventive Medicine, Faculty of Medicine, Universiti Teknologi MARA, Sungai Buloh Campus, Jalan Hospital, 47000 Sungai Buloh, Selangor Malaysia

**Keywords:** Traditional and complementary medicine, Patient assessment of chronic illness care, Metabolic syndrome, Primary care

## Abstract

**Background:**

Traditional and Complementary Medicine (TCM) is widely used particularly among patients with chronic diseases in primary care. However, evidence is lacking regarding TCM use among patients with Metabolic Syndrome (MetS) and its association with patients’ experience on chronic disease conventional care that they receive. Therefore, this study aims to determine the prevalence and pattern of TCM use, compare the patients’ experience of chronic disease care using the Patient Assessment of Chronic Illness Care - Malay version (PACIC-M) questionnaire between TCM users and non-users and determine the factors associated with TCM use among patients with MetS in primary care.

**Methodology:**

A cross-sectional study was conducted at a university primary care clinic. Patients aged 18 to 80 years old with MetS were recruited. Socio-demographic characteristic, clinical characteristics and information on TCM use and its pattern were recorded in a proforma. Patient’s experience of chronic disease conventional care was measured using PACIC-M questionnaire. The comparison of PACIC-M mean score between TCM users and non-users was measured using independent t-test. The factors associated with TCM use were determined by simple logistic regression (SLogR), followed by multiple logistic regression (MLogR).

**Results:**

Out of 394 participants, 381 (96.7%) were included in the final analysis. Of the 381 participants, 255 (66.9%) were TCM users (95% CI 62.7, 71.7). Only 36.9% of users disclosed about TCM use to their health care providers (HCP). The overall mean PACIC-M score was 2.91 (SD ± 0.04). TCM users had significantly higher mean PACIC-M score compared to non-users (2.98 ± 0.74 vs 2.75 ± 0.72, *p* = 0.01). The independent factors associated with TCM use were being female (Adj. OR 2.50, 95% CI 1.55, 4.06), having high education level (Adj. OR 2.16, 95% CI 1.37, 3.41) and having high overall PACIC-M mean score (Adj. OR 1.49, 95% CI 1.10, 2.03).

**Conclusion:**

TCM use was highly prevalent in this primary care clinic. However, the disclosure rate of TCM use to HCP was low. Females, those with high education and high PACIC-M mean score were more likely to use TCM. Further research should explore the reasons for their TCM use, despite having good experience in conventional chronic disease care.

## Background

World Health Organization (WHO) defines Traditional and Complementary Medicine (TCM) as two separate entities [1]. ‘Traditional Medicine’ (TM) is defined as “the sum total of the knowledge, skill, and practice based on the theories, beliefs, and experiences indigenous to different cultures, whether explicable or not, used in the maintenance of health, as well as in the prevention, diagnosis, improvement or treatment of physical and mental illness” [1]. Whereas ‘Complementary Medicine’ (CM) or ‘Alternative Medicine’ (AM) is defined as “a broad set of health care practices that are not part of that country’s own tradition or conventional medicine and are not fully integrated into the dominant health-care system”. The combination of terms i.e. Complementary and Alternative Medicine (CAM), is used widely and interchangeably with Traditional and Complementary Medicine (TCM) in many countries [1].

TCM is widely used around the globe especially among individuals with chronic conditions such as Metabolic Syndrome (MetS) and its components such as diabetes, hypertension, dyslipidaemia and central obesity [2]. In Malaysia, a study by Kew showed that TCM usage was higher among individuals with diabetes, hypertension and hypercholesterolaemia (31.7%) than the general population (25.9%) [3]. Approximately 20–30% of them used TCM as a substitute for their conventional medications [3]. In studies conducted by Baharom and Ching, TCM was used among patients with diabetes to complement their conventional medicines in order to achieve better diabetes control [4, 5].

Although some TCM was found to be effective in decreasing waist circumference, blood glucose, blood lipids and blood pressure, there are concern about adverse effects and complications especially when it is used simultaneously with conventional treatment [2, 6, 7]. A study by Jatau et al. has reported that TCM consumption was associated with hepatotoxicity, miscarriage, hypertensive urgency and psychiatric disorder [7]. Hence, suggestion has been made to improve the quality of conventional care to minimize the use of TCM in order to avoid complications [8].

The quality of conventional chronic disease management in primary care could be improved with the implementation of the Chronic Care Model (CCM) [9, 10]. This model consists of six interconnected elements which include healthcare organisation, delivery system design, clinical information system, decision support, patient self-management support and use of community resources [10]. The CCM emphasises on developing productive interactions between informed, actively engaged patients with proactive and prepared healthcare teams [10]. In order to measure patient’s experience in receiving conventional chronic disease care which is congruent with the CCM, the Patient Assessment of Chronic Illness Care (PACIC) questionnaire was developed [11].

In Malaysia, the integration of TCM into conventional care is currently limited to secondary health care services [12]. Integration of TCM practice in primary care is not well established. Therefore, patients who perceived that they receive better conventional care consistent with the CCM were thought to be less likely to use TCM as an alternative or a complementary to their conventional treatment in primary care. Conceptually, we hypothesized that patients with a higher PACIC mean score would be less likely to use TCM in primary care.

To the best of our knowledge, it was not known whether PACIC score would be independently associated with TCM use among patients with MetS. Therefore, the objectives of this study were to determine the prevalence and pattern of TCM usage, to compare the difference in PACIC scores between TCM users and non-users and to determine the factors associated with TCM use among patients with MetS in primary care.

## Methods

### Study design and population

A cross sectional study was conducted at a university primary care clinic in Selangor, Malaysia. A total of 381 patients aged 18 to 80 years old who fulfilled the eligibility criteria were recruited. The inclusion and exclusion criteria for this study were similar to those used in our previous study involving patients with MetS [13].

The inclusion criteria were patients who: (a) attended the primary care clinic for at least 6 months; (b) had blood investigations (fasting serum lipid [FSL], fasting plasma glucose [FPG] and haemoglobin A1c [HbA1c]) done in the past 6 months; (c) could read and understand the Malay language; (d) fulfilled at least 3 out of 5 Joint Interim Statement (JIS) 2009 diagnostic criteria for MetS [14], i.e. systolic blood pressure (BP) ≥130 mmHg and/or diastolic BP ≥85 mmHg or on treatment for hypertension (HPT); FPG ≥5.6 mmol/L or on treatment for elevated glucose; triglycerides (TG) ≥1.7 mmol/L or on treatment for dyslipidaemia; high-density lipoprotein cholesterol (HDL-C): male < 1.0 mmol/L, female < 1.3 mmol/L or on treatment for dyslipidaemia; waist circumference (WC) South Asian cut-points: male ≥90 cm, female ≥80 cm; and (e) were willing to participate in this study.

**Fig. 1:**
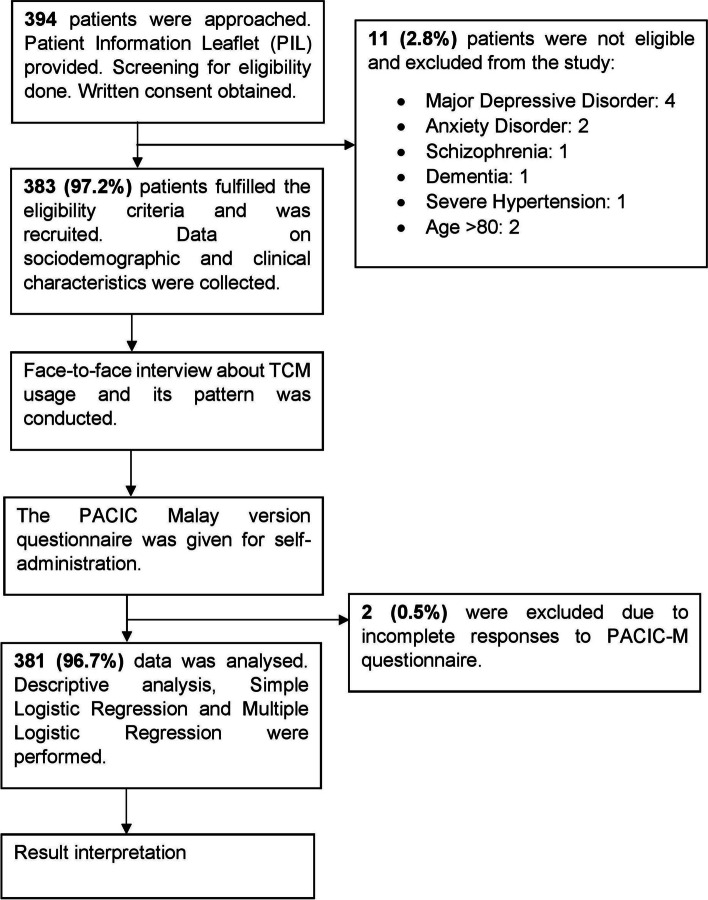
Flow chart of the conduct of the study

The following patients were excluded: (a) presented with severe HPT (systolic BP > 180 mmHg and/or diastolic BP > 110 mmHg); (b) had underlying secondary HPT; (c) diagnosed with circulatory disorders requiring secondary care over the past 1 year (e.g. unstable angina, heart attack, stroke, transient ischemic attacks, peripheral vascular disease); (d) on renal dialysis; (e) received shared care at secondary care centres; (f) enrolled in another intervention study; (g) pregnant; (h) diagnosed with malignancy; (i) had any form of mental disorders or cognitive impairments that would affect the ability to answer the questionnaire; and (j) unable to give informed consent. Figure 1 shows the flow chart of the study.

### Study tools

#### Traditional and complementary medicine (TCM) proforma

A proforma was used to gather information about TCM utilisation which comprised of a) sociodemographic characteristics; b) clinical characteristics including anthropometric measurements, blood investigation results and medical background; c) patterns of TCM use including reasons for use, source of information regarding TCM, source of TCM, types of TCM, cost spent on TCM in a year and reason for non-disclosure to medical practitioner about TCM use.

#### PACIC-M questionnaire

The Malay version of the PACIC questionnaire (PACIC-M) was used in this study to measure patients’ experience on chronic disease conventional care that they received [15]. It is valid and reliable with Cronbach’s α of 0.94 and the intra-class correlation coefficient of 0.93 [15]. This questionnaire consists of 19 items, framed within three components: a) goal setting/tailoring and problem solving/contextual; b) follow-up/coordination and c) patient activation and delivery system design/decision support [15]. Participants were required to response to at least the last 9 items of PACIC-M (item 11–19) which represent the problem solving/contextual and follow-up/coordination scales in order to be included in the analysis [11]. Each item is scored by 5-point Likert Scales from 1 (almost never) to 5 (almost always) [15]. The mean score of items from each component and the overall score across all 19 items were measured in this study [15]. A higher PACIC-M mean score represents better patient’s experience in receiving chronic disease care that aligns with the CCM [11]. Table 1 shows the 3 components of PACIC-M and the corresponding items for each component.

**Table 1:** Components of Patient Assessment of Chronic Illness Care-Malay version (PACIC-M) and items for each component

PACIC-M Component		PACIC-M Item
1.	Goal setting/tailoring and problem solving/contextual	5–14
2.	Follow-up/coordination	15–19
3.	Patient activation and delivery system design/decision support	1–4

### Definition of study variables

The dependent variable (DV) of this study was TCM utilisation which was divided into ‘TCM user’ and ‘TCM non-user’. TCM in this study was defined as one or more practices or modalities being used by the participants other than treatment provided by the current treating health care providers (HCP) [1]. The reasons for use included prevention and treatment of physical or mental illness or as part of cultural practices or religious belief [1]. The types of TCM included in this study were as categorised by the Ministry of Health (MOH), Malaysia in the National Health and Morbidity Survey (NHMS) 2015 report. There were six main TCM practices [16]. The subtypes for each main practice are shown in Table 2 [16].

**Table 2:** Types of Traditional and Complementary Medicine in Malaysia

**Types of Traditional and Complementary Medicine**	
**Traditional Malay Medicine**	
Malay Herbs	
Malay Cupping	
Malay Massage	
**Traditional Chinese Medicine**	
Chinese Herbs	
Chinese Cupping	
Acupuncture	
Tuina	
Qi Gong	
**Traditional Indian Medicine**	
Ayurveda	
Siddha	
Unani	
Yoga & Naturopathy	
**Homeopathy**	
**Islamic Medical Practice**	
**Complementary Therapy**	
Hypnotherapy	
Psychotherapy	
Reiki	
Aura Metaphysic	
Color Vibration Therapy	
Chiropractic	
Osteopathy	
Reflexology	
Complementary group of massage (Thai, Swedish, Balinese/Javanese massage)	
Aromatherapy	
Nutritional Therapy	
**Supplementary Products**	

‘Current TCM user’ was defined as a person who was using one or more types of TCM among the six main practices within the past 1 year prior to data collection [16]. ‘Past TCM user’ was defined as a person who used one or more types of TCM among the six main practices for at least once in his/her lifetime, but was no longer using within the last 1 year prior to data collection [16]. ‘Non-TCM user’ was defined as a person who never use any type of TCM in his/her lifetime [16]. In this study, the DV for ‘TCM user’ was defined as ‘current TCM user’. Meanwhile, ‘TCM non-user’ was defined as the combination of ‘non-TCM user’ and ‘past TCM user’. This is shown in Table 3.

**Table 3:** Definition of Traditional and Complementary Medicine use

No.	Category	Definition	Dependent Variable	Justification
1.	Current TCM user	A person who was using any TCM modalities within the past one year prior to data collection.	TCM user	The main variable of interest, in line with the objectives of this study.
2.	Past TCM user	A person who used TCM at least once in his/her lifetime, but was no longer using within the last one year prior to data collection.	TCM non-user	These two categories were combined. There was no significant difference in the mean age and gender distribution between these two groups.^b^
3.	Non-TCM user	A person who never use any type of TCM in his/her lifetime.		

^b^Statistically, there was no significant difference in the mean age (t = 0.60, df = 95.23, *p* = 0.57) and gender (X^2^ = 1.48, df = 1, *p* = 0.22) between ‘past TCM user’ and ‘non-TCM user’

*TCM* Traditional and Complementary Medicine, *HCP* Health care providers

The independent variables (IV) of this study were sociodemographic characteristics, clinical characteristics and PACIC-M mean score. Sociodemographic data included age, gender, marital status, ethnicity, education level, occupational status and household income group. Ethnicity was categorized based on the main ethnic groups in Malaysia which are Malay, Chinese and Indian. Education level was categorized based on the Malaysian education system which comprised of no formal education, primary education (standard 1 to 6), secondary education (Form 1 to 5) and higher tertiary education (pre-university course, diploma, degree, masters and PhD level). With regards to the household income, it was grouped based on the monthly household income categorized by the Department of Statistics, Malaysia (DOSM) in 2016 [17]. The top 20% (T20) were those who earned more than or equal to Ringgit Malaysia (RM) 9620 per month, the middle 40% (M40) were those who earned RM 4360 to RM 9619 per month and the bottom 40% (B40) were those who earned less than RM 4360 per month [17].

The clinical characteristic data included smoking status, BMI, WC, BP, FPG, TG, HDL and HbA1C levels. The BMI is categorized based on the recommended cut-off points for the Asian population which are underweight (BMI < 18.5 kg/m^2^), normal (18.5–22.9 kg/m^2^), overweight (23.0–27.4 kg/m^2^) and obese (≥27.5 kg/m^2^) [18]. The cut-off points for other clinical factors were defined according to the JIS criteria [19]. The PACIC-M score was regarded as a continuous variable where the overall mean score and the mean score for each component were calculated.

### Sample size determination

Sample size was calculated using the OpenEpi Version 3 opensource calculator for ‘Sample size for a proportion of descriptive study’ available from https://www.openepi.com/SampleSize/SSPropor.htm

The equation for the calculation was:

$$\boldsymbol{n}=\left[\mathbf{DEFF}\ast \mathbf{Np}\ \left(\mathbf{1}\hbox{-} \mathbf{p}\right)\right]/\left[\right({\mathbf{d}}^{\mathbf{2}}/{{\mathbf{Z}}^{\mathbf{2}}}_{\mathbf{1}\hbox{-} \boldsymbol{\upalpha} /\mathbf{2}}\ast \left(\mathbf{N}\hbox{-} \mathbf{1}\right)+\mathbf{p}\ast \left(\mathbf{1}\hbox{-} \mathbf{p}\right)\Big]$$

n = sample size

DEFF = design effect (for cluster surveys)

N = population size (for finite population correction factor or fpc)

p = hypothesized % frequency of outcome factor in the population

d = desired precision

*Z*^2^ 1 − α/2 = confidence interval

The population size was determined by the total number of patients registered in the electronic medical record (EMR) in the primary care clinic i.e. 10,000 in a year. The prevalence of TCM utilisation was estimated as 31.7% based on a previous community-based cross-sectional survey among adults with cardiovascular risk factors in Pahang, Malaysia [3]. By taking confidence level of 95% with the desired precision of 5%, the minimum sample required for this study was 323 participants. After considering 20% of non-eligibility and non-responder rate, we targeted to approach a total of 388 participants.

### Sampling method and participant recruitment

Participant recruitment and data collection were conducted over 14-week duration from September to December 2019. Adults aged ≥18 years old who attended the primary care clinic were approached and invited to participate in this study. Patient information leaflet was given. Those who verbally agreed to participate were screened for eligibility and written informed consent was obtained.

### Sources of data

The data for this study were obtained from several sources i.e. i) anthropometry measurements, ii) EMR for medical background and blood investigation results, iii) face-to-face interview for the sociodemographic data and TCM utilization. All of these data were transferred into the TCM Proforma. Data on patient’s experience on chronic disease care were obtained through self-administration of the PACIC-M questionnaire.

### Data collection procedure

#### Anthropometry measurement

The anthropometry measurements i.e. BP, WC and Body Mass Index (BMI) were performed by the trained nurses in the pre-treatment room. BP was measured using an automatic digital blood pressure monitor (Omron HBP-1100). The participant was advised not to smoke, exercise, or consume caffeinated beverages in the last 30 min prior to the BP measurement [20]. The participant was allowed to rest for at least 5 min before the measurements were taken [20]. The participant was seated upright with the back laid and supported [20]. The right arm was placed on the table with the upper arm at the heart level [20]. The appropriate BP cuff was placed covering two third of the right upper arm [20]. BP was measured twice with 2 min interval [20]. The average of these two BP readings was taken as the BP value for each participant [20].

WC was measured to the nearest 0.1 cm using a non-stretchable measuring tape. The measurement was taken at the midpoint between the lower rib margin and the iliac crest, while the participants standing with both arms at the side [21]. WC was measured at the end of exhalation [21].

Weight in kilogram (kg) and height in metre (m) were measured using the adult weighing scale and stadiometer (Charder MS4900). The weight was measured to the nearest 0.1 kg when the participant was standing on the scale with light clothing and without foot wear. The height was measured to the nearest 0.01 m when the participant was standing on the same scale facing forward with the back, buttock and heels against the scale. Two readings for each weight and height were measured and the average measurement was recorded. The Body Mass Index (BMI) was calculated using the formula, BMI = weight (kg)/ [height (m)]^2^.

#### Retrieval of data from electronic medical record

The participant’s medical background and the latest biochemistry investigation results were retrieved from the EMR. The investigation results included FPG, FSL (TG and HDL-C) and HbA1c taken within the last 6 months.

#### Face-to-face interview

Data on sociodemographic and TCM utilization were obtained through face-to-face interview by two research assistants. To minimize interview bias, the interviewers were trained prior to data collection to ensure consistency and standardization.

#### Questionnaire administration

The PACIC-M questionnaire was given to the participants for self-administration after the interview. A clear verbal instruction on the questionnaire answering technique was given. Participants were required to complete the questionnaire on their own without referring to any notes or their companion. The questionnaire was returned to the research assistants once completed and it was checked for completeness.

### Statistical analysis

Descriptive analyses were used to describe the socio-demographic characteristics, clinical characteristics, proportion of TCM use, TCM pattern, overall PACIC-M score and score of its components. Normality of the data was assessed using histogram, Kolmogorov-Smirnov and Shapiro-Wilk tests for continuous variables. If the data were normally distributed, they were described using mean and standard deviation (SD). If the data were not normally distributed, they were described using median and interquartile range (IQR). Categorical variables were described in numbers and percentages.

For comparison of PACIC-M score between ‘TCM users’ and TCM non-users’, the normality of data distribution and equality of variance was examined accordingly. Data for the overall PACIC-M score and the score for each of the component were normally distributed. Therefore, independent t-test was used to compare the difference in overall mean PACIC-M score and the mean score for each component between ‘TCM users’ and ‘TCM non-users’.

Simple Logistic Regression (SLogR) analysis was used to screen the association between the independent variables (sociodemographic data, clinical characteristics, overall PACIC-M score and score of each component) with TCM utilisation. Variables with a *P*-value of < 0.25 from the SLogR analysis were then included in the Multiple Logistic Regression (MLogR) analysis [22]. Stepwise forward and backward binary logistic regressions were performed. Confounders were adjusted in the MLogR using stepwise selection procedure. The underlying assumption for this approach is that all potential confounders would be selected and included into the regression model [23]. Model fitness was checked using the Hosmer-Lemeshow goodness-of-fit test. Interactions, multicollinearity, and assumptions were also checked. The best fit regression model was chosen as the final model for this study. Statistical significant was taken at a *P*-value of < 0.05 [22]. The data were analysed using the IBM SPSS Statistics software version 23.

## Results

### Recruitment of participants

A total of 394 patients were approached and screened for eligibility. Eleven patients (2.8%) were excluded from the study due to non-eligibility and two participants were excluded from the analysis due to incomplete response to PACIC-M questionnaire. Therefore, 381 (96.7%) participants were included in the final analysis. This is shown in Fig. 1.

### Sociodemographic and clinical characteristic

The sociodemographic and clinical characteristics of the 381 participants in this study are shown in Table 4. The mean age was 59.4 years old (SD ± 8.4). More than half of them were male (58%), Malays (87.1%), had high education level (53.8%), and retired (53.5%). Less than half belonged to the low-income group (42.8%).

**Table 4:** Sociodemographic and clinical characteristic of the study participants (*N* = 381)

Variable	Non-TCM user, ***n*** = 54	Past TCM user, ***n*** = 72	Current TCM user, ***n*** = 255	Total ***N*** = 381
**Age, years (n,%)**				
18–29	1 (0.3)	0 (0)	0 (0)	1 (0.3)
30–39	1 (0.3)	2 (0.5)	5 (1.3)	8 (2.1)
40–49	7 (1.8)	4 (1.0)	28 (7.3)	39 (10.2)
50–59	9 (2.4)	24 (6.3)	94 (24.7)	127 (33.3)
60–69	24 (6.3)	34 (8.9)	107 (28.1)	165 (43.3)
70–80	12 (3.1)	8 (2.1)	21 (5.5)	41 (10.8)
Mean (±SD)^b^	61.1 (±10.6)	60.1 (±8.2)	58.9 (±8.4)	59.4 (±8.4)
**Gender**^b^**(n,%)**				
Male	40 (10.5)	46 (12.1)	135 (35.4)	221 (58.0)
Female	14 (3.7)	26 (6.8)	120 (31.5)	160 (42.0)
**Marital Status (n,%)**				
Unmarried	7 (1.8)	9 (2.4)	19 (5.0)	35 (9.2)
Married	47 (12.3)	63 (16.5)	236 (61.9)	346 (90.8)
**Ethnicity (n,%)**				
Malay	45 (11.8)	64 (16.8)	223 (58.5)	332 (87.1)
Chinese	3 (0.8)	4 (1.0)	15 (3.9)	22 (5.8)
Indian	6 (1.6)	3 (0.8)	12 (3.1)	21 (5.5)
Others	0 (0.0)	1 (0.3)	5 (1.3)	6 (1.6)
**Educational Level (n,%)**				
No formal education	0 (0.0)	0 (0.0)	2 (0.5)	2 (0.5)
Primary	5 (1.3)	7 (1.8)	7 (1.8)	19 (5.0)
Secondary	26 (6.8)	36 (9.4)	93 (24.4)	155 (40.7)
Tertiary	23 (6.0)	29 (7.6)	153 (40.2)	205 (53.8)
**Occupational Status (n,%)**				
Unemployed	4 (1.0)	10 (2.6)	35 (9.2)	49 (12.9)
Employed	14 (3.7)	25 (6.6)	89 (23.4)	128 (33.6)
Retiree	36 (9.4)	37 (9.7)	131 (34.4)	204 (53.5)
Household Income per Month* (n,%)				
B40 (<RM 4360)	31 (8.1)	35 (9.2)	97 (25.5)	163 (42.8)
M40 (RM 4360–9619)	15 (3.9)	28 (7.3)	96 (25.2)	139 (36.5)
T20 (>RM 9619)	8 (2.1)	9 (2.4)	62 (16.3)	79 (20.7)
**Smoking Status (n,%)**				
Smoker	6 (1.6)	13 (3.4)	28 (7.3)	47 (12.3)
Non-smoker	48 (12.6)	59 (15.5)	227 (59.6)	334 (87.7)
**Body Mass Index, kg/m**^**2**^**(n,%)**				
Underweight/Normal (< 22.9)	0 (0.0)	2 (0.5)	13 (3.4)	15 (3.9)
Overweight (23.0–27.4)	23 (6.0)	21 (5.5)	61 (16.0)	105 (27.6)
Obese (≥27.5)	31 (8.1)	49 (12.9)	181 (47.5)	261 (68.5)
Mean (±SD)	29.3 (±4.7)	29.7 (±4.7)	30.2 (±5.1)	30.0 (±4.9)
**Waist Circumference, cm (n,%)**				
Normal (male < 90, female < 80)	5 (1.3)	3 (0.8)	7 (1.8)	15 (3.9)
Abnormal (male ≥90, female ≥80)	49 (12.9)	69 (18.1)	248 (65.1)	366 (96.1)
Median (IQR)	96.0 (15.0)	97.0 (14.0)	97.0 (10.0)	97.0 (11.0)
**Systolic Blood Pressure, mmHg (n,%)**				
Normal (< 130)	6 (1.6)	17 (4.5)	56 (14.7)	79 (20.7)
Abnormal (≥130)	48 (12.6)	55 (14.4)	199 (52.2)	302 (79.3)
Mean (±SD)	146.8 (±14.7)	140.2 (±14.3)	139.8 (±14.7)	140.8 (±14.8)
**Diastolic Blood Pressure, mmHg (n,%)**				
Normal (< 85)	39 (10.2)	53 (13.9)	193 (50.7)	285 (74.8)
Abnormal (≥85)	15 (3.9)	19 (5.0)	62 (16.3)	96 (25.2)
Mean (±SD)	78.7 (±10.0)	77.9 (±9.4)	77.3 (±10.0)	77.6(± 9.9)
**Triglyceride, mmol/L (n,%)**				
Normal (< 1.7)	31 (8.1)	42 (11.0)	171 (44.9)	244 (64.0)
Abnormal (≥1.7)	23 (6.0)	30 (7.9)	84 (22.0)	137 (36.0)
Median (IQR)	1.6 (0.9)	1.5 (0.9)	1.4 (0.9)	1.5 (1.0)
**High-Density Lipoprotein, mmol/L (n,%)**				
Normal (male ≥1.0, female ≥1.3)	45 (11.8)	59 (15.5)	202 (53.0)	306 (80.3)
Abnormal (male < 1.0, female < 1.3)	9 (2.4)	13 (3.4)	53 (13.9)	75 (19.7)
Mean (±SD)	1.3 (±0.3)	1.2 (±0.2)	1.3 (±0.3)	1.3 (± 0.3)
**Fasting Plasma Glucose**^a^**, mmol/L (n,%)**				
Normal (< 5.6)	17 (5.7)	18 (6.0)	69 (23.1)	104 (34.8)
Abnormal (≥ 5.6)	26 (8.7)	41 (13.7)	128 (42.8)	195 (65.2)
Median (IQR)	5.7 (1.8)	5.9 (2.2)	5.8 (1.7)	5.8 (1.8)
**HbA1c**^c^**, % (n,%)**				
Controlled (< 6.5)	5 (4.2)	4 (3.3)	20 (16.7)	29 (24.2)
Uncontrolled (≥ 6.5)	12 (10.0)	20 (16.7)	59 (49.2)	91 (75.8)
Mean (±SD)	7.7 (±1.4)	8.1 (±1.7)	7.4 (±1.6)	7.6 (± 1.6)

*Based on the Report of Household Income and Basic Amenities Survey 2016 by Department of Statistics, Malaysia

^a^Missing value, no result available (*n* = 82)

^c^Missing value, no result available for patients without diabetes (*n* = 261)

^b^Statistically, there was no significant difference in the mean age (t = 0.60, df = 95.23, *p* = 0.57) and gender (X^2^ = 1.48, df = 1, *p* = 0.22) between ‘past TCM user’ and ‘non-TCM user’

Majority of the participants were non-smokers (87.7%), obese (68.5%), had abnormal WC (96.1%), abnormal systolic BP (79.3%), abnormal FPG (65.2%) and uncontrolled HbA1c (75.8%).

### Traditional and complementary medicine utilisation

#### Prevalence of traditional and complementary medicine

**Fig. 2:**
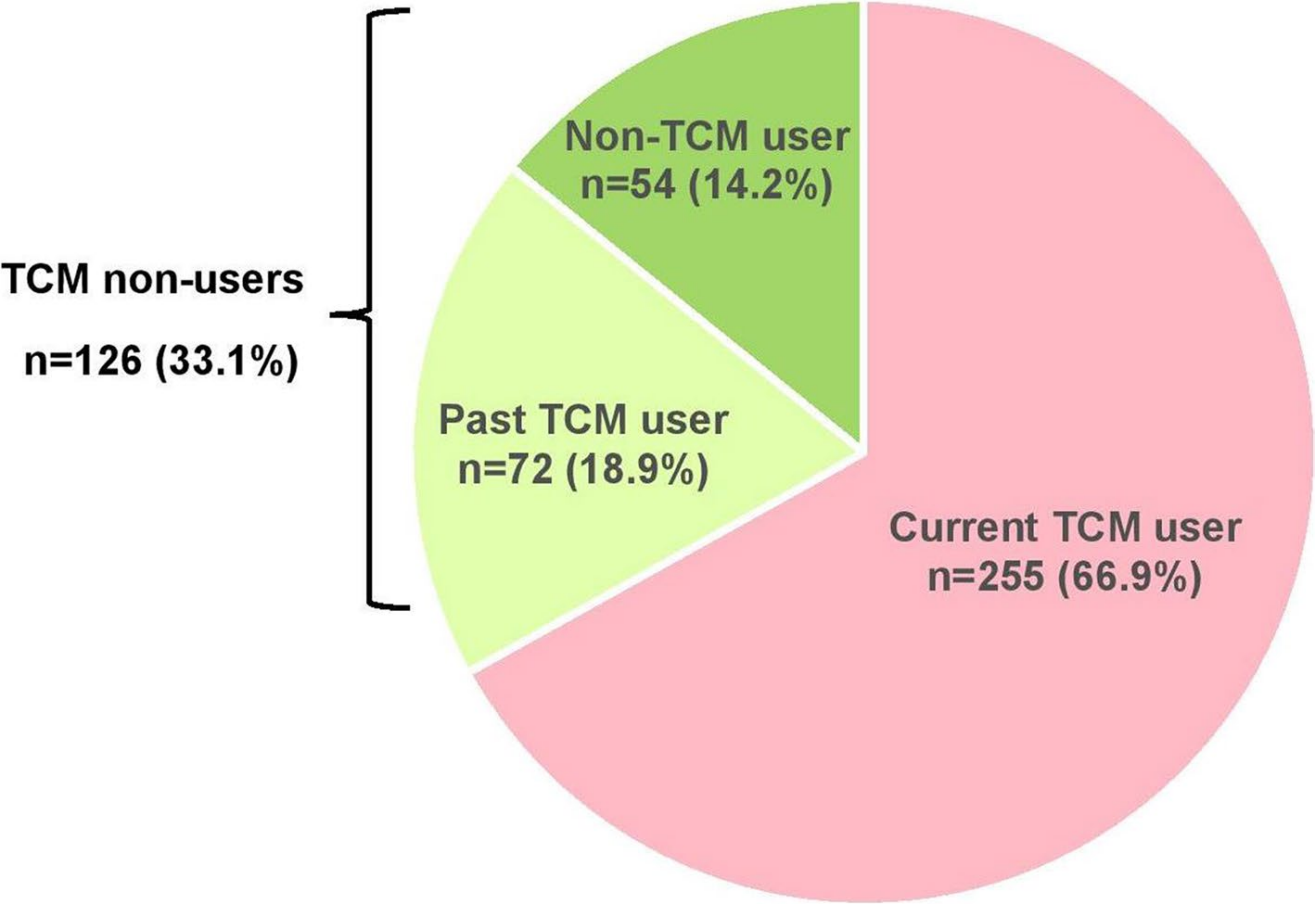
Distribution of participants according to Traditional and Complementary Medicine utilisation (*N* = 381)

Out of 381 participants, 255 (66.9%) were TCM users (95% CI 62.7, 71.7), and 126 (33.1%) were non-users (95% CI 28.3, 37.3). The prevalence of TCM use is shown in Fig. 2.

#### Reasons for traditional and complementary medicine use

Out of 255 TCM users, 47.8% (95% CI 41.6, 54.3) used TCM to maintain wellness, 26.3% (95% CI 20.7, 31.8) used it for therapeutic purposes and 22.4% (95% CI 17.3, 27.4) used it for both wellness and therapeutic reasons. Among those who were using TCM for therapeutic purposes, 84.2% used it as complementary to the conventional medicine. These findings are shown in Table 5.

**Table 5:** Reason and pattern of Traditional and Complementary Medicine utilisation among users (*n* = 255)

Pattern of use	Frequency, n (%)
**Reasons for TCM Use**	
Maintain wellness only	122 (47.8)
Therapeutic purpose only	67 (26.3)
Both wellness and therapeutic	57 (22.4)
Religious reason	9 (3.5)
**Sources of Information**^a^	
Friends	108 (30.0)
Family	79 (21.9)
Others	53 (14.7)
Social Media	44 (12.2)
Health care providers	37 (10.3)
Mass Media	28 (7.8)
TCM providers	11 (3.1)
**Sources of TCM**^a^	
Pharmacy	112 (33.0)
TCM User’s house	80 (23.6)
TCM Kiosk	77 (22.7)
TCM providers	53 (15.7)
Online	9 (2.7)
Health facilities	8 (2.4)
**Types of TCM**^a^	
Health Supplement	124 (47.5)
Traditional Malay Medicine	85 (32.6)
Other Complementary Medicine	23 (8.8)
Traditional Chinese Medicine	14 (5.4)
Islamic Medical Practice	8 (3.1)
Homeopathy	4 (1.5)
Traditional Indian Medicine	3 (1.1)
**Cost, RM (per month)**	
Min-Max	0–4000
Mean ± SD	127.9 ± 16.7
Median ± IQR	100.0 ± 100
**Disclosure to Health Care Provider**	
No	161 (63.1)
Yes	94 (36.9)
**Reasons for Non-Disclosure**^a^	
Never asked by HCP	126 (72.8)
Not important to disclose	32 (18.5)
Others	15 (8.7)

^a^Multiple response analysis

#### Patterns of traditional and complementary medicine utilisation

In this study, majority of the users obtained the information regarding TCM from friends (30%) and family members (21.9%). Among all the users, 33% obtained the TCM from the pharmacies. The majority of TCM users were using health supplements (47.5%) followed by Traditional Malay Medicine (32.6%). With regards to expenditure, the mean spending for TCM was RM 127.9 (SD ± 16.7) per month. These are shown in Table 5.

#### Traditional and complementary medicine disclosure

Out of 255 TCM users, only 36.9% disclosed the information on their TCM use to the HCP. Majority of the participants did not reveal the information because they were never asked by the treating HCP (72.8%). These are also shown in Table 5.

### PACIC-M score

The overall mean score for all participants was 2.91 (±0.04). TCM users had significantly higher overall mean score of 2.98 (±0.74) compared to the non-users, 2.75 (±0.72). Regarding each component of the PACIC-M, the mean scores were significantly higher in TCM users compared to non-users for all the components. These are shown in Table 6.

**Table 6:** The comparison of PACIC-M mean score between TCM users and non-users (*N* = 381)

PACIC-M Score	Mean (±SD)			T^a^ (df)	Mean Difference (95% CI)^a^	***P***-value*
	Total ***N*** = 381	Non-user ***n*** = 126	User ***n*** = 255			
Overall Score	2.91 (0.04)	2.75 (0.72)	2.98 (0.74)	−2.86 (379)	−0.23 (− 0.39, − 0.71)	**0.005**
Component 1: Goal setting/tailoring and problem solving/contextual	3.06 (0.84)	2.93 (0.86)	3.13 (0.83)	−2.14 (379)	−0.20 (− 0.38, − 0.02)	**0.033**
Component 2: Follow-up/coordination	2.11 (0.94)	1.93 (0.87)	2.20 (0.95)	−2.67 (379)	−0.27 (− 0.47, − 0.07)	**0.008**
Component 3: Patient activation and delivery system design/decision support	3.51 (0.84)	3.33 (0.87)	3.60 (0.82)	−2.91 (379)	−0.26 (− 0.44, − 0.09)	**0.004**

*statistically significant at *p* ≤ 0.05

^a^statistical test: independent t-test

### Factors associated with traditional and complementary medicine use

Out of eleven potential independent variables included in the MLogR analysis, three variables were found to be independently associated with TCM use, which were being female (Adj. OR 2.50, 95% CI 1.55, 4.06), having high education level (Adj. OR 2.16, 95% CI 1.37, 3.41) and having high overall PACIC-M mean score (Adj. OR 1.49, 95% CI 1.10, 2.03). Hosmer-Lemeshow goodness-of-fit test showed that the final model was fit (*p* = 0.475). Classification table showed overall percentage of 69.3% indicating that the model could correctly predict 69.3% whether they were TCM user or not. The receiver operating characteristic (ROC) curve gave an area under the curve (AUC) of 0.659 (95%CI 0.601, 0.717) which indicated that the model could accurately discriminate 65.9% of the samples. The results from the SLogR and final MLogR analysis are shown in Tables 7 and 8.

**Table 7:** Factors associated with TCM use among patients with Metabolic Syndrome from SLogR analysis

Variable	B (S.E.)	Wald (df)^a^	***P***-value*	Crude OR (95% CI)
**Age in Years**	−0.02 (0.01)	2.92 (1)	**0.087**	0.98 (0.95,1.00)
**Gender**				
Male				1.00
Female	0.65 (0.23)	8.01 (1)	**0.005**	1.91 (1.22, 2.99)
**Marital Status**				
Unmarried (single/widower/divorcee)				1.00
Married	0.59 (0.36)	2.72 (1)	**0.099**	1.81 (0.90, 3.65)
**Ethnicity**				
Non-Malay				1.00
Malay	−0.08 (0.32)	0.07 (1)	0.796	0.92 (0.49, 1.73)
**Educational Level**				
Low Education (no formal education/primary/secondary)				1.00
High Education (tertiary)	0.76 (0.22)	11.72 (1)	**0.001**	2.14 (1.38, 3.30)
**Occupational Status**				
Unemployed/Retiree				1.00
Employed	−0.18 (0.23)	0.59 (1)	0.443	1.84 (0.53, 1.32)
**Household Income per Month**				
B40 (<RM 4360)				1.00
M40 (RM 4360–9619)	0.42 (0.24)	2.96 (1)	**0.086**	1.52 (0.94, 2.45)
T20 (>RM 9619)	0.91 (0.32)	8.23 (1)	**0.004**	2.48 (1.33, 4.62)
**Smoking Status**				
Smoker				1.00
Non-smoker	0.36 (0.32)	1.30 (1)	0.254	1.44 (0.77, 2.69)
**Body Mass Index, kg/m**^**2**^	0.03 (0.23)	1.33 (1)	0.249	1.03 (0.98, 1.07)
**Waist Circumference, cm**				
Normal (male < 90, female < 80)				1.00
Abnormal (male ≥90, female ≥80)	0.88 (0.53)	2.74 (1)	**0.098**	2.40 (0.85, 6.78)
**Systolic Blood Pressure, mmHg**				
Normal (< 130)				1.00
Abnormal (≥130)	−0.23 (0.28)	0.70 (1)	0.402	0.79 (0.46, 1.36)
**Diastolic Blood Pressure, mmHg**				
Normal (< 85)				1.00
Abnormal (≥85)	−0.14 (0.25)	0.32 (1)	0.572	0.87 (0.53, 1.41)
**Triglyceride, mmol/L**				
Normal (< 1.7)				1.00
Abnormal (≥1.7)	−0.39 (0.22)	3.03 (1)	**0.082**	0.68 (0.44, 1.05)
**High-Density Lipoprotein, mmol/L**				
Normal (male ≥1.0, female ≥1.3)				1.00
Abnormal (male < 1.0, female < 1.3)	0.22 (0.28)	0.59 (1)	0.443	1.24 (0.72, 2.15)
**Fasting Plasma Glucose, mmol/L**				
Normal (< 5.6)				1.00
Abnormal (≥ 5.6)	−0.03 (0.26)	0.15 (1)	0.903	0.97 (0.59, 1.60)
**HbA1c, %**				
Controlled (≤6.5)				1.00
Uncontrolled (> 6.5)	−0.19 (0.45)	0.17 (1)	0.683	0.83 (0.34, 2.03)
**Overall PACIC-M Mean Score**	0.43 (0.15)	7.87 (1)	**0.005**	1.53 (1.14, 2.06)
**Component 1 PACIC-M Mean Score**	0.28 (0.13)	4.49 (1)	**0.034**	1.32 (1.02, 1.70)
**Component 2 PACIC-M Mean Score**	0.33 (0.13)	6.89 (1)	**0.009**	1.39 (1.09, 1.78)
**Component 3 PACIC-M Mean Score**	0.38 (0.13)	8.16 (1)	**0.004**	1.46 (1.13, 1.89)

*statistically significant at α ≤ 0.25

^a^statistical test: simple logistic regression

**Table 8:** Independent factors associated with TCM use among patients with Metabolic Syndrome from MLogR analysis

Variable	Adjusted B (S.E.)	Wald (df)^a^	***P***-value*	Adjusted OR (95% CI)
**Age**	−0.02 (0.15)	1.46 (1)	0.227	0.98 (0.96, 1.01)
**Gender**				
Male				1.00
Female	0.91 (0.25)	13.62 (1)	**< 0.001**	**2.50 (1.55, 4.06)**
**Marital Status**				
Unmarried				1.00
Married	0.75 (0.39)	3.77 (1)	0.052	2.12 (0.99, 4.52)
**Education Level**				
Low education level				1.00
High education level	0.80 (0.23)	11.06 (1)	**0.001**	**2.16 (1.37, 3.41)**
**Household Income**				
B40				1.00
M40	0.10 (0.28)	0.13 (1)	0.722	1.10 (0.64, 1.91)
T20	0.52 (0.37)	1.97 (1)	0.161	1.65 (0.81, 3.49)
**Waist Circumference**				
Normal				1.00
Abnormal	0.68 (0.58)	1.40 (1)	0.237	1.97 (0.64, 6.10)
**Triglycerides**				
Normal				1.00
Abnormal	−0.46 (0.25)	3.49 (1)	0.062	0.63 (0.39, 1.02)
**Component 1 PACIC-M Mean Score**	−0.78 (0.63)	1.53 (1)	0.216	0.46 (0.13, 1.58)
**Component 2 PACIC-M Mean Score**	−0.10 (0.29)	0.11 (1)	0.740	0.91 (0.52, 1.60)
**Component 3 PACIC-M Mean Score**	−1.23 (1.30)	0.90 (1)	0.343	0.29 (0.99, 1.78)
**Overall PACIC-M Mean Score**	0.40 (0.16)	6.31 (1)	**0.011**	**1.49 (1.10, 2.03)**

*statistically significant at α ≤0.05

^a^statistical test: multiple logistic regressions. The Hosmer-Lemeshow goodness-of-fit test showed the final model was fit (*P* = 0.475). There were no interaction or multicollinearity problems

## Discussion

To the best of our knowledge, this is the first study to establish the association between TCM use with patient’s experience of chronic disease care measured by the PACIC-M score. Our study also adds to the literature regarding prevalence and pattern of TCM use among patients with MetS in primary care.

The prevalence of TCM use among patients with MetS in our primary care clinic was higher (66.9%) compared to a previous local community-based study among patients with multiple cardiovascular risk factors i.e. diabetes, hypertension and dyslipidaemia (31.7%) [3] and also compared to a study in the UK (55.5%) [24]. Despite the high prevalence, only 36.9% of TCM users in our study disclosed the use of TCM to their HCP. Majority (72.8%) of them did not disclose the information due to lack of inquiry from medical providers. Some patients (18.5%) perceived that it was not important to disclose TCM use to their HCP. The low rate of disclosure and the reasons of non-disclosure in our study are similar to the findings in a previous systematic review and meta-analysis [25]. This is concerning as non-disclosure of TCM use to the HCP can be harmful as TCM may interact with conventional medicine [7, 26, 27]. Hence, it is vital for HCP to enquire about TCM use and educate patients regarding the need to disclose information on their TCM use.

With regards to the patients’ experience on conventional chronic disease care, our study shows that the overall PACIC-M mean score among patients with MetS in our primary care setting was satisfactory (2.91 ± 0.04). Previous studies have reported various ranges of PACIC scores depending on the study locality [28–31]. The variations in PACIC scores observed in different countries may be influenced by the differences in health care delivery system and population background such as socio-economics and cultural factors [28]. Furthermore, the use of translated versions of PACIC questionnaire in various studies might produce different results [28]. When comparing TCM users and non-users, our study shows that TCM users were found to have significantly higher PACIC-M mean score (2.98 ± 0.74) compared to the non-users (2.75 ± 0.72). However, direct comparison with other studies could not be made as there was no study which has explored PACIC score among TCM users.

Several factors have been found to be associated with TCM use in this study, which were being female, having high education level and having high overall PACIC-M mean score. In our study, women were found to be twice more likely to use TCM compared to men. Our finding is similar to studies in India and Turkey [32, 33]. Women have been found to have better help-seeking behaviours compared to men [34] and are thought to be more involved in self-care and self-treatment [35], and this could be the reason for their high TCM use. Generally, women are more likely to use any form of health care [36] including TCM [37]. Other reasons of TCM use among women in other studies were to cater for low self-esteem and body image concerns as part of their self-improvement [38]. However, our finding is in contrast with a previous study among selected rural communities in Malaysia, which has found that men were more likely to use TCM [39]. Meanwhile, a narrative review concluded that several studies did not demonstrate any association between gender and TCM use [36].

With regards to education, our study has found that those with higher education were twice more likely to use TCM. Similar finding has been observed in previous studies conducted in Malaysia [39, 40] and Europe [41]. A systematic review also found that high education level was one of the most common predictors of TCM use [42]. Individuals with higher education had better awareness and ability to search for information about TCM [36], which probably explains the reason for their higher usage. They were more likely to explore TCM usage to find a satisfactory combination of TCM and conventional medicine [41]. In contrast to the finding in our study, several other studies in other parts of the world demonstrated that lower education status was associated with TCM use [43].

With regards to patients’ experience on conventional chronic disease care, our study found that those with higher PACIC-M mean score were 1.5 times more likely to use TCM. Our study included patients with MetS who are under regular follow-up in our primary care clinic and majority of them used TCM as complementary to the conventional medicine. Although they have good experience in conventional chronic disease care, they still preferred to complement their conventional treatment with TCM. This is in contrast with our hypothesis that patients with a higher PACIC mean score would be less likely to use TCM in primary care. The finding in our study calls for further qualitative research to explore the reasons of high TCM usage despite them having a good experience in conventional chronic disease care. This could be driven by differences in health beliefs or poor disease control, which are beyond the scope of our study.

### Strengths and limitations

The strength of this study included its novel findings in establishing the association between patient’s experience in receiving chronic disease care and TCM use in a primary care setting. This addresses the knowledge gap in this area which was previously under-explored.

This study has several limitations. First, the use of convenience sampling method in this study may be prone to sampling bias. However, efforts were made to reduce this bias as patients were approached and invited consecutively on the designated data collection day. Secondly, this study was conducted at a university primary care clinic located in an urban area where both conventional medicine and TCM are easily accessible. Thus, the findings may not be generalised to other primary care settings especially the rural areas where the population and availability of conventional medicine and TCM might be different. Thirdly, majority of our participants were Malays which explained the finding in which the use of Traditional Malay Medicine was highly prevalent. Finally, this study did not include other potential factors that may influence TCM use such as patient-physician relationship, health literacy, perceived effectiveness and side effects of conventional medicine and TCM. Therefore, the findings from the logistic regression analysis were only limited to the variables included in this study.

### Implications to clinical practice and further research

TCM use was highly prevalent among patients with MetS in this study. However, the rate of disclosure to HCP was low due to lack of inquiry from the providers and also the perception that disclosure is unimportant. Therefore, HCP should routinely enquire regarding TCM use, especially among patients with multiple cardiovascular risk factors such as MetS. HCP should also empower themselves with evidence-based knowledge on the effectiveness and safety of various types TCM in order to counsel their patients appropriately. TCM use in Malaysia should also be better regulated to ensure efficacy and safety.

Further research should include qualitative studies to explore reasons for TCM use among patients with MetS. It should also be conducted in other primary care settings in both rural and urban areas with various ethnic populations such as Chinese and Indians, to improve generalisability. Further studies should also include other potential factors that may influence TCM use.

## Conclusion

In conclusion, the findings of this study call for HCP to routinely enquire about TCM use among patients with MetS and counsel them appropriately as the disclosure rate was low and the prevalent was high. Being female, having high education and better patient’s experience in chronic disease care were identified as the independent factors associated with TCM use. Further research is needed to explore the reasons of high TCM usage despite them having a good experience in conventional chronic disease care.

## Data Availability

Data are kept at the Primary Care Medicine Department, Universiti Teknologi MARA (UiTM), Selayang Campus, Jalan Prima Selayang 7, 68100 Batu Caves, Selangor, Malaysia. Data will be shared by the corresponding author upon request and it is subjected to the data protection regulations.

## Abbreviations

AM: Alternative medicine
BMI: Body mass index
BP: Blood pressure
CAM: Complementary and alternative medicine
CCM: Chronic Care Model
EMR: Electronic medical record
FPG: Fasting plasma glucose
JIS: Join Interim Statement
MeTS: Metabolic syndrome
MLogR: Multiple logistic regression
NHMS: National Health and Morbidity Survey
PACIC: Patient Assessment of Chronic Illness Care
RM: Ringgit Malaysia
ROC: Receiver operating characteristic
SD: Standard deviation
SLogR: Simple logistic regression
SPSS: Statistical Package for Social Sciences
TCM: Traditional and complementary medicine
TM: Traditional medicine
WC: Waist circumference
WHO: World Health Organization
